# Systematic evaluation of different doses of cyclophosphamide induction therapy for lupus nephritis

**DOI:** 10.1097/MD.0000000000009408

**Published:** 2017-12-22

**Authors:** Ming Tian, Xiaohong Song, Liping Dong, Xing Xin, Junwu Dong

**Affiliations:** a Department of Nephrology, Puai Hospital; b Department of Obstetrics and Gynecology, Tongji Hospital, Tongji Medical College, Huazhong University of Science and Technology, Wuhan, Hubei, P.R.China.

**Keywords:** cyclophosphamide, lupus nephritis, meta-analysis, randomized controlled trial, systemic evaluation

## Abstract

**Objective:**

This study systemically evaluated the efficacy and safety of intermittent intravenous pulse therapy with different doses of cyclophosphamide (CTX) for the treatment of lupus nephritis (LN).

**Methods:**

We screened the Chinese Journal Full-text Database (CNKI, 1994–present), China Biology Medicine (CBMdisc, 1978–present), VIP Database for Chinese Technical Periodicals (1989–present), PubMed (1948–present), MEDLINE (Ovid SP, 1946–present), Embase (1947–present), and the Cochrane controlled trials register (13, 2017). Literature reports were selected according to the inclusion and exclusion criteria, effective data were extracted, research quality was evaluated, and RevMan5.2 was used for meta-analysis.

**Results:**

Seven randomized controlled studies were included, consisting of 655 patients. The meta-analysis results showed no significant differences between the low- and high-dose cyclophosphamide groups in partial, complete, and total remission rates as well Systemic Lupus Erythematosus Disease Activity Index (SLEDAI). Furthermore, there were no significant differences between the 2 groups in hematologic toxicity and gastrointestinal reaction, but the risk of infection (risk ratio [*RR*] = 0.74, 95% confidence interval [CI], 0.56–0.98, total effect inspection *Z* = 2.12, *P* = .03), and menstrual disorder (*RR* = 0.46, 95% CI, 0.31–0.69, total effect inspection *Z* = 3.83, *P* = .0001) decreased in the low-dose cyclophosphamide group.

**Conclusions:**

There was no obvious difference between the low- and high-dose cyclophosphamide groups in efficacy in the treatment of lupus nephritis, but the risk of infection and menstrual disorder significantly decreased in the low-dose group.

## 1 Introduction

Systemic lupus erythematosus (SLE) is a chronic progressive autoimmune disease involving systemic multisystem and is often accompanied by renal lesions, called lupus nephritis (LN) with higher morbidity and mortality.^[1]^ A study showed that kidney damage occurs in up to 60% of patients with lupus. In addition, approximately 10% to 15% of patients with LN progress to end-stage renal disease and require blood dialysis to sustain life, whereas the 5-year survival rate is approximately 82%.^[2]^Therefore, early diagnosis and control of the development of LN are the keys to improving the prognosis of patients and improving survival.^[3]^ Intermittent intravenous pulse therapy with high-dose cyclophosphamide (CTX) combined with glucocorticoid has been a classic treatment for severe LN with an obvious improvement of survival rate since the early 1980s when it was used clinically.^[4]^

However, this treatment often leads to numerous adverse reactions including leukopenia, infection, reproductive toxicity, hair loss, and gastrointestinal reactions. Furthermore, the immunosuppression induced by CTX has a slow onset with obvious time and dose dependency.^[5]^ The gradually increasing survival rate of patients with LN has led to the proposal of a higher safety requirement for long-term medication. In recent years, numerous clinical studies have shown that intermittent intravenous pulse therapy with low-dose CTX combined with glucocorticoid has a superior efficacy in the treatment of LN with less adverse reactions than high-dose regimens do.^[6–8]^ To the best of our knowledge, our study is the first to investigate the efficacy and safety of different doses of CTX in the treatment of LN using a meta-analysis.

## 2 Materials and methods

All procedures performed in this study were in accordance with the ethical guidelines of the ethics committee of our institution (Tongji Hospital, Tongji Medical College, Huazhong University of Science and Technology), the national research committee, and the 1964 Helsinki Declaration and its later amendments or comparable ethical standards.

## 3 Inclusion criteria

The types of research studies included in this analysis were randomized controlled trials (RCTs) with no matter allocation concealment or blinding methods, and the publication language was not limited. The research objects were, no limitation on age, sex, and race and conducted in accordance with the SLE and LN diagnostic criteria of the American Rheumatology Association.^[9]^ For the interventions, the treatment and control groups were administered low- and high-dose CTX, respectively, whereas doses, as well as the use of hormones and other immunosuppressive agents, were similar in both groups (referring to the corresponding literature). Treatment course was ≥6 months.

## 4 Exclusion criteria

The published studies excluded from this analysis were non-RCT studies, RCT studies that adopted self-controlled research analysis, duplicated publications, and reports including only abstracts without the full text.

## 5 Efficacy evaluation

The parameters analyzed for the efficacy evaluation were the partial, complete, and total remission rates, as well as the systemic lupus erythematosus disease activity index (*SLEDAI*). It is noteworthy that the specific evaluation standard of the partial, complete, and total remission rates varied between the analyzed literature reports, and was calculated according to the unified standard based on the system evaluators used in this study.

## 6 Safety evaluation

The safety evaluation involved the analysis of infection, blood system toxicity, gastrointestinal reactions, and menstrual disorders.

## 7 Database for literature retrieval

The following databases were thoroughly searched over the indicated time periods to retrieve the relevant literature reports: Chinese Journal Full-text Database (CNKI, 1994–present), China Biology Medicine (CBMdisc, 1978–present), VIP Database for Chinese Technical Periodicals (1989–present), PubMed (1948–present), MEDLINE (Ovid SP, 1946–present), Embase (1947–present), and the Cochrane controlled trials register (13, 2017). The following keywords were used in the search fields: Chinese: “lupus nephritis” and “cyclophosphamide” and English: “lupus nephritis,” “lupus glomerulonephritis,” “proliferative glomerulonephritis,” “membranous glomerulonephritis,” and “cyclophosphamide.” References of the relevant literature were also reviewed to supplement any research that may have been missed.

## 8 Data extraction

Each clinical research study was selected and evaluated by 2 evaluators independently. The title and abstract of the literature reports were read, and those that did not meet the inclusion criteria were excluded, whereas the included studies were reviewed for the complete test. A unified data extraction table was used to extract the following information: general information: title, authors’ names, publication date, and source of literature; research characteristics: the general situation of the research subjects and the intervention measures; and measurement indexes: such as outcome.

## 9 Quality evaluation

A bias risk assessment was carried out on all the included literature using the Cochrane system manual (version 5.0).^[10]^ The evaluation content included: blinding of participants, personnel, and outcome assessment; random sequence generation; incomplete outcome data; selective reporting; allocation concealment; and other biases. Furthermore, each item was designated as low- or high-bias risks, as well as unclear for the degree of bias risk. Disagreements over the assessments were resolved by discussions between the evaluators, or a third evaluator was involved when required. Relevant information not provided in the research report was obtained by contacting the original authors.

## 10 Statistical analysis

The RevMan 5.2 was used to perform the statistical analysis.^[11,12]^ The dichotomy data were represented using the relative risk (RR) and 95% confidence interval (CI), and differences with a *P* < .05 were considered statistically significant. The included research studies were subjected to a clinical heterogeneity evaluation, when this was not detected, a χ^2^ test (*P* < .1, for significant heterogeneity) was used for the qualitative analysis and the *I*^2^ test for quantitative analysis (*I*^2^ < 25%, 25% < *I*^2^ < 50%, and *I*^2^ > 50% for mild, moderate, and high heterogeneity, respectively). In the absence of statistical heterogeneity between the studies, the fixed-effect model was used, whereas in cases of heterogeneity, the randomized effect model was used.

## 11 Results

### 11.1 Literature retrieval

**Figure 1:**
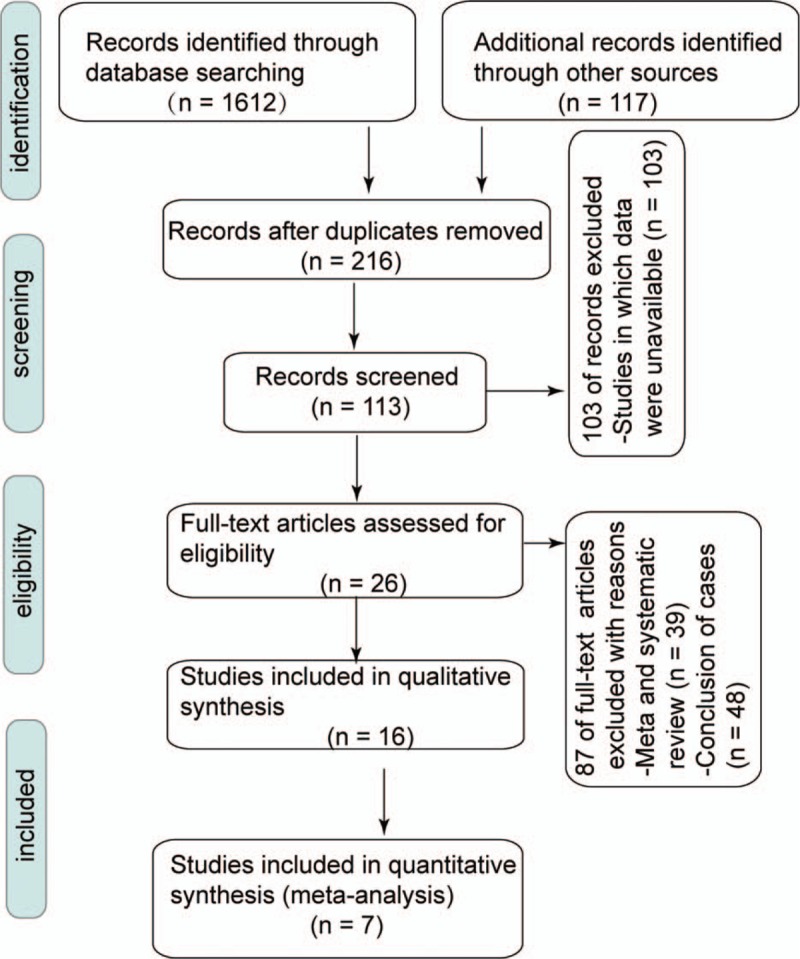
Preferred reporting items for systematic reviews and meta-analyses (PRISMA) flow chart of study selection.

A total of 1729 literature reports were retrieved, and after screening using the inclusion and exclusion criteria, 7 studies fulfilled the requirements including 2 Chinese and 5 English language reports. Furthermore, 655 patients were included, as shown in Figure 1.

### 11.2 Research characteristics

All the studies were published between 2002 and 2017 and consisted of one multicenter and 6 single-center studies. A total of 655 patients were included (307 and 348 cases from the low- and high-dose CTX treatment groups, respectively). The largest and smallest sample sizes were 225 and 40 cases, respectively (low- and high-dose CTX treatment: 18–113 and 22–112, respectively). The literature report characteristics are shown in Table 1.

**Table 1:**
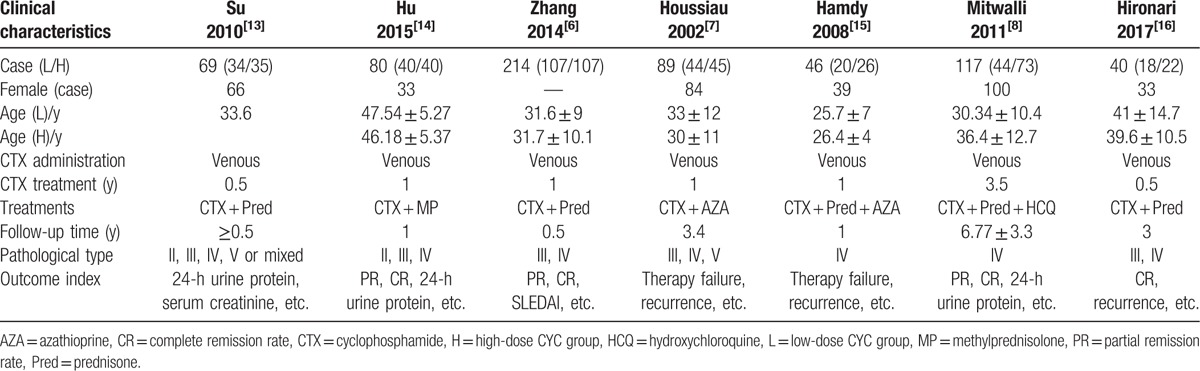
Basic characteristics of each study in the meta-analysis.

### 11.3 Quality evaluation of included literature

**Figure 2:**
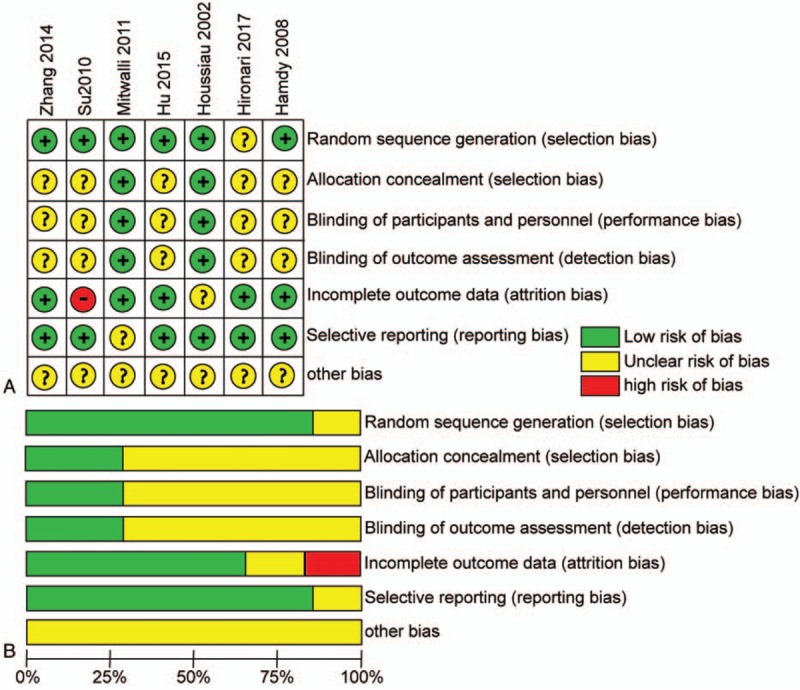
Risk of bias (A) summary and (B) graph: review of authors’ judgments about the risk of each bias item for each included study presented as percentages across all included studies.

Six of the literature reports used random grouping, and the methods were explained. All 7 included literature reports described the number of patients who were withdrawn or lost in the follow-up visits in detail. A total of 25 cases were withdrawn or lost in the follow-up visits. The quality evaluation of each literature report is shown in Figure 2.

## 12 Meta-analysis

### 12.1 Efficacy evaluation of the different doses of CTX in LN treatment

Three of the 7 included studies reported the partial remission rate, and there was a lower heterogeneity among these studies than the other studies (*P* = .92, *I*^2^ = 0%). Therefore, the fixed-effects model was adopted for the analysis, and the results showed no significant statistical differences occurred between the groups in the partial remission rate (*RR* = 1.08, 95% CI, 0.89–1.32, total effect inspection *Z* = 0.79, *P* = .43). This observation suggests that the partial remission rate was similar between the high- and low-dose CTX induction therapy for LN.

**Figure 3:**
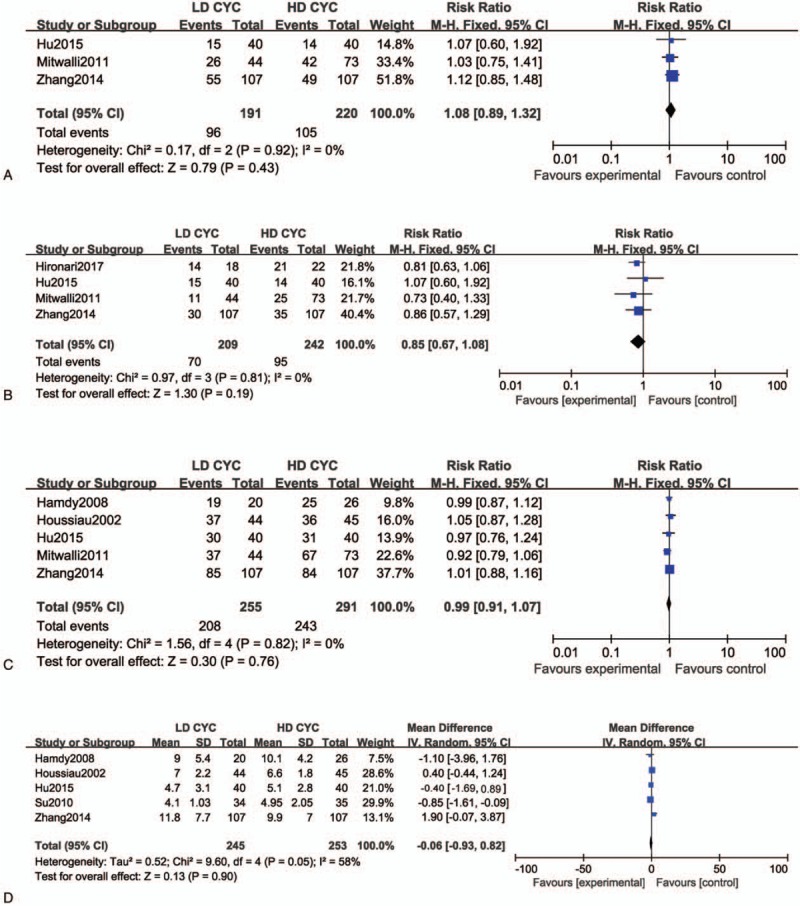
Forest plot of risk ratio (RR) and 95% confidence intervals (CIs) of evaluated efficacy of different cyclophosphamide (CTX) doses for treatment of lupus nephritis (LN). (A) Partial, (B) complete, and (C) total remission rates. (D) Systemic lupus erythematosus disease activity index (SLEDAI). Squares represent the RR of each study, and the area of each square was proportional to the weight of each study in the meta-analysis; horizontal lines, 95% CIs; closed diamond, pooled RR with their 95% CIs.

Four articles included the complete remission rate, and there was a lower heterogeneity among these studies (*P* = .81, *I*^2^ = 0%) than among the other studies and, so, the fixed-effects model was adopted for the analysis. The results showed that no significant statistical differences occurred in both groups in the complete remission rate (*RR* = 0.85, 95% CI, 0.67–1.08, total effect inspection *Z* = 1.30, *P* = .19). This result suggests that the complete remission rate was similar between the high- and low-dose CTX induction therapy for LN. In addition, 5 articles included the total remission rate, and there was a lower heterogeneity among these studies (*P* = .82, *I*^2^ = 0%) than in the other studies. Therefore, the fixed-effects model was adopted for analysis, and the results showed that no significant statistical differences occurred between the groups in the complete remission rate (*RR* = 0.99, 95% CI, 0.91–1.07, total effect inspection *Z* = 0.30, *P* = .76). These observations suggest that the total remission rate between the high- and low-dose CTX induction therapy for LN. In addition, 5 articles included the SLEDAI score and there was a high heterogeneity among these studies (*P* = .05, *I*^2^ = 58%). Therefore, the randomized effects model was adopted for the analysis and the results showed no significant statistical differences between the groups in the SLEDAI scores after induction therapy (*RR* = −0.06, 95% CI, −0.93 to 0.8, total effect inspection *Z* = 0.13, *P* = .90). This observation suggests that the remission rate was similar between the high- and low-dose CTX induction therapy for LN (Fig. 3).

### 12.2 Safety evaluation of CTX treatment for LN

#### 12.2.1 Infection

The heterogeneity among the studies was low (*P* = .28, *I*^2^ = 20%) and, therefore, the fixed-effect model was adopted for the safety evaluation. The results showed that compared with the high-dose CTX induction therapy, the low-dose had a significantly lower incidence of infection caused by LN (*RR* = 0.74, 95% CI, 0.56–0.98, total effect inspection *Z* = 2.12, *P* = .03).

#### 12.2.2 Hematologic toxicity

The heterogeneity among the studies was low (*P* = .43, *I*^2^ = 0%) and, therefore, the fixed-effect model was adopted. The results showed no significant statistical differences between the groups in hematotoxicity (*RR* = 0.68, 95% CI, 0.41–1.15, total effect inspection *Z* = 1.43, *P* = .15).

#### 12.2.3 Gastrointestinal reaction

The heterogeneity among the studies was high (*P* = .002, *I*^2^ = 83%) and, therefore, the randomized effect model was adopted. The results showed no significant statistical differences between the groups in gastrointestinal reactions (*RR* = 0.46, 95% CI, 0.12–1.72, total effect inspection *Z* = 1.16, *P* = .25).

#### 12.2.4 Menstrual disorders

**Figure 4:**
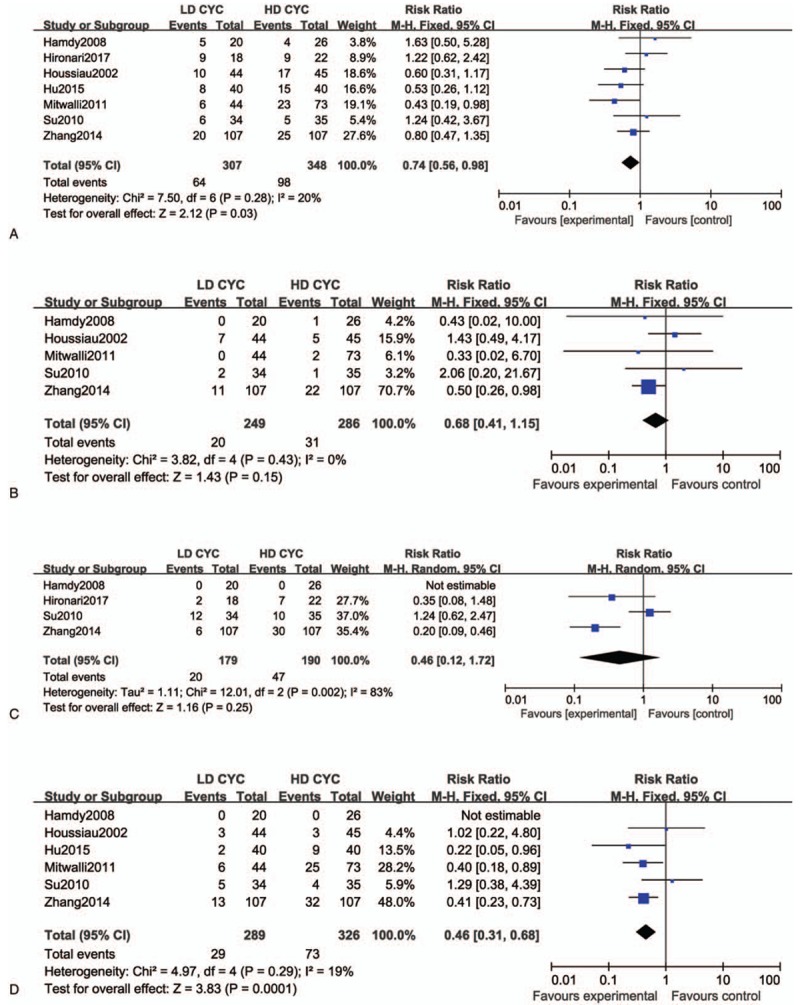
Forest plot of risk ratio (RR) and 95% confidence intervals (CIs) of evaluated safety cyclophosphamide (CTX) treatment for lupus nephritis (LN). (A) infection, (B) hematotoxicity, (C) nausea or vomiting, and (D) menstrual disorders. Squares represent the RR of each study, and the area of each square was proportional to the weight of each study in the meta-analysis; horizontal lines, 95% CIs; closed diamond, pooled RR with their 95% CIs.

The heterogeneity among the studies was low (*P* = .29, *I*^2^ = 19%) and, therefore, the fixed-effect model was adopted. The results showed there was a lower risk of menstrual disorder with the low-dose CTX induction therapy than with the high-dose therapy for LN (*RR* = 0.46, 95% CI, 0.31–0.69, total effect inspection *Z* = 3.83, *P* = .0001; Fig. 4).

## 13 Discussion

The renal injury associated with SLE gradually progresses from the early mild lesions to glomerular sclerosis and, subsequently, leads to end-stage renal disease, and is also one of the main causes of death in patients with SLE.^[17]^ LN has numerous histological and clinical characteristics. Currently, it is the accepted practice to perform a kidney biopsy as a standard reference to confirm the type of nephritis and obtain information related to the treatment and prognosis of the disease.^[18,19]^ The pathological types of LN^[20,21]^ include class I: minimal mesangial LN, class II: mesangial proliferative LN, class III: focal LN (<50% glomeruli), class IV: diffuse LN (>50% glomeruli), class V: membranous LN, and class VI: advanced sclerosing LN.

Studies have shown that compared with patients with LN who were partly relieved or unrelieved, those with complete alleviation had a better clinical prognosis and, so, it is critical to initiate induction therapy that achieves complete alleviation.^[22,23]^ This is especially important for those with class III, IV, V, or a combination (III + V or IV + V) who need aggressive immunosuppressive therapy to achieve remission of the active inflammatory process and reduce the probability of relapses and long-term renal failure.^[24]^ Beginning in the 1970s, the National Institutes of Health (NIH)^[25–27]^ carried out a series of clinical RCTs on CTX treatment of SLE. The results showed that the intermittent intravenous infusion CTX pulse therapy was superior to a single application of prednisone in controlling the progress of kidney diseases inducing remission and protecting renal function. Thus, a foundation was laid for the use of CTX as an important drug for SLE and determined the NIH standards. The induction phase involves the intravenous infusion of CTX once every month, 6 or 7 times. Furthermore, 500 to 1000 mg/m^2^ (body surface area) of CTX or combined with a venous drip of methylprednisolone or daily oral administration of hormones. The maintenance phase included the intravenous infusion of CTX once every 3 months, continuously for 2 years or another 1 year after remission. However, there are obvious associated side effects such as secondary infection, bone marrow suppression, and menstrual disorders, which are often dose and time dependent. Longer medication regimens and larger doses lead to earlier and severer adverse reactions.^[28]^ Recently, many researchers have proposed an improved low-dose CTX regimen,^[29–32]^ which is safer with an equivalent efficacy to that of the high-dose CTX treatment. However, these results are based on small sample size studies, which lack confirmation by large-scale clinical RCTs.

According to the screening criteria, 7 RCT literature reports were selected, which reported venous pulse CTX doses of 500 to 1000 mg/m^2^ and 400 to 500 mg in the high- and low-dose groups, respectively. Furthermore, 6 and 7 literature reports were included in the efficacy assessment and safety analysis, respectively. The results showed that the partial, complete, and total remission rates, as well as the SLEDAI scores, were comparable in patients with LN who were induced with the high- and low-dose CTX. Furthermore, regarding safety, infections and leukopenia have always been the major limiting factor in lupus therapy,^[33,34]^ but in the present study, the risk of infection and menstrual disorder was significantly lower in LN patients on the low-dose CTX group than the high-dose group.

In addition, the incidence of basic bone marrow suppression and gastrointestinal reactions was the same in the 2 groups. The results were consistent with a recent retrospective study of a single central low-dose CTX inductive therapy for patients with type III/IV/V LN.^[35]^ In addition, research studies have investigated low-dose CTX, tacrolimus, and mycophenolate mofetil induction therapy for patients with type III and type IV LN. There were no obvious statistical differences among 3 groups in safety and efficacy.^[16]^ This suggests that low-dose CTX induction therapy may be more suitable LN treatment.

There are some limitations to this meta-analysis such as the studies included were few, and some were not high quality. The included subjects and the pathological types were not consistent, and there were differences in the responses of the different pathological types of LNs and race to the drug.^[36]^ The induction regimens of each study were not identical such as the CTX + AZA vs CTX + MP. CTX dose was decreased because of the reduction in white blood cells during the induction period, which could be affected by the clinical efficacy discrimination. The efficacy and safety evaluation indexes differed among the studies. The study was based on short-term efficacy and safety comparison. Therefore, high-quality, large-scale, multicenter RCTs with a longer follow-up would be needed to further compare the safety and effectiveness.

CTX is an alkylating agent, which has been widely used in the treatment of autoimmune disease such as LN because of its strong immunosuppression, but its potential carcinogenic risk, reproductive toxicity, and other side effects limit its use. Presently, there is no consensus on the treatment course and dosage of CTX in the treatment of LN. The findings of this study suggest that intermittent intravenous pulse therapy with low-dose CTX (400–500 mg) is safer and effective in treating LN, and should be promoted clinically. Furthermore, large-scale RCTs are needed in the future to guide the precise use of CTX.

## Acknowledgments

We thank for all the patients who are involved in the studies selected in this meta-analysis.
